# Multiple Micronutrients, Lutein, and Docosahexaenoic Acid Supplementation during Lactation: A Randomized Controlled Trial

**DOI:** 10.3390/nu12123849

**Published:** 2020-12-16

**Authors:** Ella Schaefer, Hans Demmelmair, Jeannie Horak, Lesca Holdt, Veit Grote, Karoline Maar, Christoph Neuhofer, Daniel Teupser, Nadja Thiel, Erwin Goeckeler-Leopold, Silvia Maggini, Berthold Koletzko

**Affiliations:** 1 Bayer Consumer Care AG, Peter-Merian-Straße 84, 4002 Basel, Switzerland; silvia.maggini@bayer.com; 2 Department Paediatrics, Division Metabolic and Nutritional Medicine, Dr. von Hauner Children’s Hospital, LMU University Hospitals, Ludwig-Maximilians-Universität Munich, Lindwurmstraße 4, 80337 Munich, Germany; Hans.Demmelmair@med.uni-muenchen.de (H.D.); Jeannie.Horak@med.uni-muenchen.de (J.H.); Veit.Grote@med.uni-muenchen.de (V.G.); Berthold.Koletzko@med.uni-muenchen.de (B.K.); 3 Institute of Laboratory Medicine, LMU University Hospitals, Ludwig-Maximilians-Universität Munich, Marchioninistraße 15, 81377 Munich, Germany; lesca.holdt@med.uni-muenchen.de (L.H.); daniel.teupser@med.uni-muenchen.de (D.T.); 4 Gynecology & Obstetrics Specialist, Schloßstraße 2, 13507 Berlin, Germany; maar@dr-maar.de; 5 Gynecology & Obstetrics Specialist, Marienplatz 4, 85354 Freising, Germany; neuhofer@gyn-freising.de (C.N.); thiel@gyn-freising.de (N.T.); 6 Gynecology & Obstetrics Specialist, Jakobistraße 2, 59494 Soest, Germany; egoleo@gmx.de

**Keywords:** docosahexaenoic acid, lactation, lutein, maternal biomarkers, micronutrients

## Abstract

Breastfed infants require an adequate supply of critical nutrients for growth, tissue functions, and health. Recommended intakes for several nutrients are considerably higher in lactating than non-lactating women but are not always met with habitual diets. We report a randomized, double-blind clinical trial in 70 healthy lactating women in Germany evaluating the effects of supplementation with multiple micronutrients, lutein, and docosahexaenoic acid (DHA) compared to placebo on maternal nutrient status and milk composition. The primary endpoint was the effect on the change of human milk DHA content (as a proportion of total milk fatty acids) during 12 weeks of supplementation. Maternal blood and milk biomarkers were measured as secondary endpoints. Supplementation increased maternal milk DHA by 30% compared to a decline in the placebo group. Supplementation also increased maternal blood DHA (17%), eicosapentaenoic acid (4%), 25-OH-vitamin D (24%), vitamin B12 (12%), lutein (4%), and beta carotene (49%), while homocysteine decreased. No significant difference in the number of adverse events was observed between supplementation and placebo groups. In conclusion, multi-micronutrient supplementation was safe and increased maternal blood and milk concentrations of selected nutrients in healthy women.

## 1. Introduction

Breastfeeding is recommended as the optimal way of infant feeding to support healthy growth and development of the child [1]. For the first few months of life, the infant’s energy and nutrient needs should be provided by breastfeeding [2]. However, the composition of breastmilk can vary depending on the metabolic and nutritional state of the mother, the stage of lactation, and even the time of day (diurnal fluctuations) [3,4]. Although maternal dietary intake does not seem to affect breastmilk concentrations of certain micronutrients (such as calcium, iron, copper, zinc), it can induce variations in others (e.g., thiamin, riboflavin, vitamins B6, B12, A, and D, folic acid, selenium, iodine, and docosahexaenoic acid, DHA) [5].

It is important to ensure that infants have an adequate supply of critical nutrients that are important for tissue functions and health [6,7,8]. In the case of DHA, a low supply in human milk is undesirable since DHA supply in infancy has been linked to DHA accumulation in the brain [9,10] and to visual, cognitive, and immune development [11,12,13,14]. The concentration of DHA in human milk is strongly influenced by the dietary DHA intake of the mother, with the main dietary source being oily fish [15,16,17,18], and it varies widely within and among different populations [18,19]. Another relevant nutrient is lutein, a carotenoid found in the macular region of the retina that appears to affect retinal development [20]. It also preferentially accumulates in the infant brain and has been proposed to modulate cognitive function in the elderly, possibly via antioxidant mechanisms [21]. As humans are unable to synthesize lutein, a breastfed infant’s supply is dependent on maternal supply via breastmilk [22].

The recommended dietary intakes for DHA and many micronutrients are considerably higher in lactating women than in non-lactating and non-pregnant women [5]. If the maternal diet is poor during lactation, both the mother and child will be at increased risk of an inadequate nutrient status [23,24]. Increasing dietary intake of critical nutrients through selection of foods naturally rich in or fortified with such nutrients is a preferred strategy for achieving an adequate supply. However, consuming a healthy diet is not always achieved (e.g., availability of nutrient-rich foods, seasonal variations, poor dietary choices, cultural factors). Multiple micronutrient deficiencies are prevalent in both low- and high-income countries [24]. In fact, infants are among the age groups at highest risk of poor micronutrient status [25].

Although there are reasons to choose nutrient supplementation during lactation, few data on effects are available—particularly in women with no overt nutritional deficiencies [24]. Only limited data from controlled clinical trials are available on the effects of supplementation with multiple micronutrients during lactation [24]. Therefore, we carried out a randomized, double-blind, placebo-controlled trial in healthy lactating women from a high-income country to evaluate the effects of multiple micronutrient supplementation (MMS) on maternal nutrient status and milk composition. The underlying hypothesis was that supplementation might help to improve maternal blood and milk DHA and carotenoid status in a healthy population of breastfeeding women.

## 2. Materials and Methods

### 2.1. Trial Design

**Figure 1:**
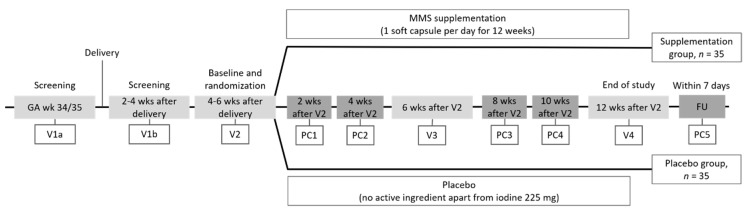
Study design. V1a, first screening; V1b, second screening; V2, randomization and baseline; V3, milk and blood sampling in mothers and measurement of infant anthropometric parameters; V4 (end of study). Phone calls to lactating women carried out between V2 and V4, final follow-up call after V4. FU, follow-up; GA, gestational age; MMS, multiple micronutrients, lutein, and docosahexaenoic acid supplementation; PC, phone call; V, visit; wks, weeks.

This multicenter, parallel, randomized, double-blind, placebo-controlled clinical trial was performed in three sites in Germany. The study was designed to compare the effects of supplementation with multiple micronutrients, lutein, and DHA provided once per day (hereafter referred to as MMS) versus placebo during lactation in healthy mothers. Supplementation began 4–6 weeks after delivery (randomization) and lasted for about 12 weeks. Five visits and five phone calls were conducted during the trial, from screening to final follow-up (Figure 1 and Supplementary Table S1). Visit 1a (first screening): healthy pregnant women were screened for study eligibility and blood was collected for safety parameters. Visit 1b (second screening): all eligibility criteria were reconfirmed, blood was collected for safety parameters, and adverse events (AEs) were assessed. Visit 2 (randomization and baseline): all eligibility criteria were reconfirmed in women and infants, after which eligible women were randomized equally to one of the two study groups and instructed to take a study capsule once daily for 12 weeks. Blood and milk from mothers were collected for baseline efficacy parameters and milk nutritional composition, respectively. Mothers’ baseline nutritional status was assessed using a food frequency questionnaire (FFQ) [26], while fatigue was assessed via a validated psychological multidimensional assessment of fatigue (MAF) questionnaire [27,28]. Anthropometric parameters in infants (weight and length) were also measured, as were concomitant medications and AEs. Visit 3 (MMS or placebo): milk and blood sampling in mothers, to assess efficacy and safety parameters, as well as assessment of their nutritional status (FFQ), wellbeing (MAF), concomitant medications, and AEs—infant anthropometric parameters were also measured. Visit 4 (end of study): mothers returned to the site for milk and blood sampling to analyze safety and efficacy parameters, while their nutritional status (FFQ), wellbeing (MAF), concomitant medications, and AEs were also recorded, and assessment of infant anthropometric evaluation took place. Phone calls to lactating women were carried out between Visits 2 and 4 to ask for compliance and breastfeeding status, with a final follow-up call after Visit 4 to check for mothers’ health status as well as stabilization of AEs and onset of new AEs.

The study protocol was reviewed by the ethics committee of the Bavarian Chamber of Physicians, Munich, Germany: EC study reference no. 16073). The trial was conducted in accordance with the Declaration of Helsinki and in compliance with all current Good Clinical Practice guidelines, local laws, and regulations. The trial was registered at ClinicalTrials.gov (ClinicalTrials.gov Identifier: NCT04462939).

### 2.2. Study Population

Healthy, pregnant, Caucasian women aged 18–45 years were screened if they were in their third trimester and expected to give birth to a healthy, singleton, full-term infant. Women were included in the study if they had a hemoglobin level > 105 g/L, intended to breastfeed for at least four months (no more than one bottle or 10% of total milk intake daily as formula), followed an omnivorous diet, did not intend to take supplements containing multivitamins, DHA, or lutein after giving birth, except for supplementation with iodine and iron, and provided written informed consent for participation in the study. Infants were included if they had reached full term (gestational age > 37 weeks < 43 weeks) and a birth weight adequate for their gestational age, had an Apgar score > 7 at 5 min after birth, and no indication of abnormal neurodevelopment.

Women were excluded if they had any clinically relevant physical, hematological, or routine laboratory abnormalities, had an infection at screening or randomization, suffered from any current metabolic (e.g., diabetes, hypothyroidism) or malabsorption disorders (e.g., chronic inflammatory bowel disease, iron accumulation or utilization disorders), had any history or current neurological, cardiac, endocrine, or bleeding disorders, or followed a restrictive diet (e.g., vegan, vegetarian, or gluten-free diet). Infants were excluded if they had congenital anomalies, gastrointestinal or metabolic disorders, perinatal hypoxia, preterm birth, or a very low birth weight (small for gestational age). Detailed inclusion and exclusion criteria are listed in Supplementary Table S2.

### 2.3. Randomization to Study Product

At baseline (Visit 2), women who fulfilled the eligibility criteria were randomized (1:1 ratio) to the MMS or placebo group. Randomization was performed using a predetermined randomization scheme generated by the Independent Unit at the Contract Research Organization (CRO), to ensure treatment blinding to study participants, investigators, and their staff, and all those involved in data collection and data analysis prior to the locking of the database.

The supplementation group was provided with oral MMS soft gel capsules (Elevit Breastfeeding and Postnatal Care, Bayer, Basel, Switzerland), which contain amounts of 12 vitamins, five minerals, lutein, and DHA close to recommended dietary intakes (Supplementary Table S3). The control group received a soft gel capsule placebo, which contained no active ingredients apart from iodine (225 μg). Iodine was included because German Maternity guidelines [29] recommend pregnancy and post-delivery iodine supplementation. Accordingly, the placebo contained the same dose of iodine (225 μg) as the MMS. Women were advised to take one capsule per day with water or another beverage from 4 to 6 weeks after delivery (Visit 2, baseline and randomization) until the end of the study (Visit 4; approximately 12 weeks of supplementation).

### 2.4. Parameters Assessed

The primary objective was the effect of maternal supplementation on the change of human milk DHA content (as a proportion (%) of total milk fatty acids) over time during supplementation. Secondary objectives included the effects on maternal blood and milk biomarkers of nutrient status, maternal fatigue, infant anthropometric parameters, and safety and tolerability.

Efficacy parameters assessed (Supplementary Table S4) included maternal blood biomarkers (i.e., the concentration of various nutrients), milk nutrient composition, maternal weight, height, and body mass index (BMI), fatigue (using the MAF questionnaire [27,28]), and nutritional status (FFQ) [26]), as well as the exploratory infant variables of weight and length, feeding, and health history. In addition, infant weight and length z-scores by age were calculated based on the World Health Organization (WHO) growth standards [30].

Safety and tolerability were assessed by evaluating the incidence and severity of AEs in mothers and infants and their relationship to trial supplementation. Laboratory parameters, physical examination, and vital signs were also recorded.

#### Biochemical Analyses

Blood was collected in plain and ethylenediaminetetraacetic acid (EDTA)-coated Monovetts (Sarstedt, Germany) to obtain serum and plasma samples respectively, by refrigerated centrifugation at 1500× *g* for 10 min. Sample aliquots were frozen at −80 °C within 2 h after collection and kept frozen until analysis. Milk samples were obtained by pumping (Medela Swing Maxi^TM^ or Medela Swing^TM^, Medela, Germany) one full breast and subsequent freezing of aliquots at −80 °C.

Glycerophospholipid fatty acids in plasma and total fatty acids in milk were analyzed from 100 and 20 µL aliquots respectively, using previously published gas chromatographic flame ionization detection (GC-FID) methods [31,32]. Homocysteine was analyzed employing a modified version of the published method by Helmuth et al. [33]. For quantitative analysis, an amino acid mixture containing 0.5 mM homocysteine (Sigma-Aldrich, Munich, Germany) and d8-homocystine (Recipe Chemicals and Instruments GmbH, Munich, Germany) was used. The calibration solutions had the concentrations 50, 25, 10, 5, 2.5, 0.5. 0.25, and 0.05 µM in water. Sample preparation was performed in 0.5 mL 96-well plates (Agilent, Waldbronn, Germany) by addition of 20 µL dithiothreitol (DTT; 77 mg/mL water; freshly prepared and kept on ice), 20 µL d8-homocystine (3.6 µM), and 20 µL plasma sample, quality control (pool of all plasma samples; 6×) or homocysteine control plasma CP1 and CP2 (Recipe, Munich, Germany). The foil-covered 96-well plate was shaken for 20 min at 400 rpm at 25 °C prior to the addition of 140 µL methanol (LC-MS grade, Merck, Darmstadt, Germany) for protein precipitation, incubation at 4 °C for 30 min, and centrifugation at 25 °C and 4000 rpm for 20 min. After pipetting 80 µL of the reduced sample into Axygen 96-well PCR plates (200 µL), 20 µL DTT solution was added and the mixture was immediately analyzed on an Agilent 1260 HPLC coupled with a Sciex QTRAP 4000 ESI-MS system. LC-MS analyses were performed according to Hellmuth et al., [33] using a silica hybride column Cogent Diamond Hybride (150 × 2.1 mm, 4 µm) (MicroSolv Technology Corporation, Leland, NC, USA). For sample analysis, Analyst 3.6 (Sciex, Darmstadt, Germany) was used, and for data evaluation, MultiQuant 3.0 (Sciex, Darmstadt, Germany) was used.

Measurements of serum folic acid, vitamin B12, and 25-OH-vitamin D were performed using electrochemiluminescense immunoassays (cobas 8000, Roche).

Measurements of carotenoids (lutein, zeaxanthin, beta-cryptoxanthin, lycopene, carotene), as well as of alpha-tocopherol and retinol in milk and blood were measured using high-performance liquid chromatography (HPLC) at SYNLAB pharma institute, Germany.

### 2.5. Power Calculation and Statistical Analyses

The primary efficacy endpoint was the change in the DHA percentage of total milk fat from baseline (Visit 2) to the end of the study (Visit 4). For the calculation of statistical power, we assumed a treatment difference of 0.15 (standard deviation (SD) 0.195) between supplement and placebo (as observed by Jensen [34]), and 28 subjects per arm were needed to achieve 80% power. To account for an assumed drop-out rate of 20%, we aimed to randomize 70 subjects (35 per arm).

After study completion, data plausibility check, and lock of the database, the primary efficacy analysis was performed on the per protocol (PP) population (all subjects with efficacy data for the primary efficacy endpoint at Visit 4 who did not have a protocol violation, i.e., milk sample not taken at Visits 2 and/or 4, which was relevant for the determination of the primary endpoint). Results were corroborated using data from the intent-to-treat (ITT) population (i.e., all subjects in the safety population who had at least one post-baseline measurement of efficacy data). The safety population comprised all subjects who were randomized into the study and took at least one dose of the study product.

For primary and secondary efficacy endpoints, analysis of covariance (ANCOVA) with supplementation as the fixed effect and baseline as a covariate was performed. Missing values were imputed using the last observation carry forward (LOCF) approach [35]. Safety and tolerability variables were assessed by evaluating incidence and severity of AEs, their relationship to trial treatment, and the incidence of abnormal findings in measurement of objective tolerability through vital signs (blood pressure, pulse rate), physical examination, and clinical laboratory findings. Only treatment-emergent AEs (TEAEs) were analyzed, i.e., AEs that began or worsened after randomization.

The statistical analysis was performed using SAS version 9.4 (SAS Institute, Inc., Cary, NC, USA) and Stata 15.1 (StataCorp, College Station, TX, USA). Continuous data are summarized as mean ± SD or LS means (least squares means) of change from Visit 2 (95% confidence interval, CI), as appropriate, while categorical data are presented by absolute and relative frequencies (*n* and %). In addition, the effect of the intervention on infant weight and length at 4 months of age was assessed using linear regression with adjustment for the respective baseline anthropometric measure at Visit 2, site, and sex, using robust standard errors assuming dependencies of values by site (cluster of site). All statistical tests were two-sided at the significance level of 0.05.

## 3. Results

### 3.1. Subject Characteristics

**Figure 2:**
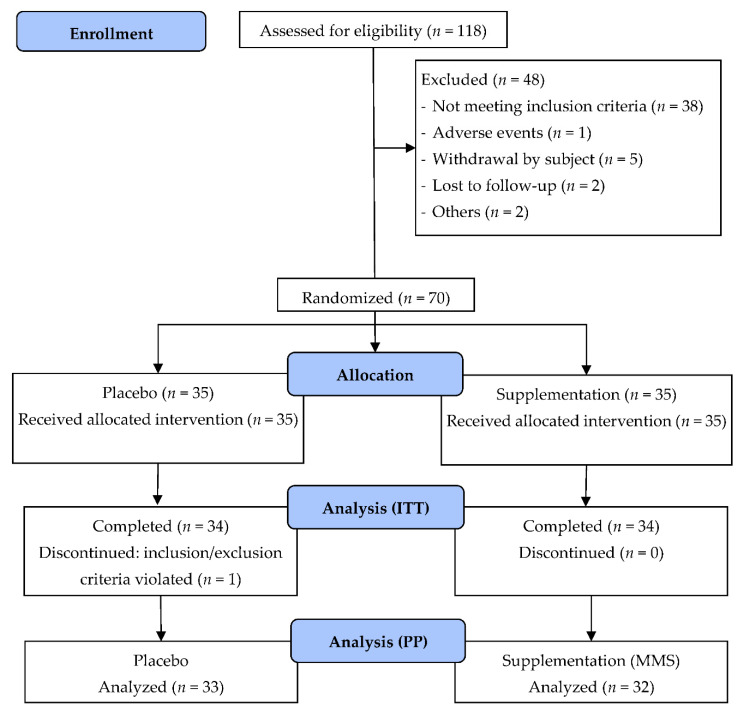
Flow diagram for study participants. ITT, intention-to-treat; MMS, multiple micronutrients, lutein, and docosahexaenoic acid (DHA) supplementation.

The clinical study was performed between 26 April 2017 and 12 November 2019. Of the 118 subjects assessed for eligibility, 70 were randomized to treatment (35 in each group), all of whom formed the safety evaluation population. Data for the primary efficacy endpoint was available in 65 subjects, who formed the per protocol population (MMS group, *n* = 32; placebo group, *n* = 33) (Figure 2). Both groups were similar at baseline (Table 1). The mean age was 31.7 ± 3.8 (21–39) years, 93.8% were Caucasian, and the mean BMI was 25.0 ± 3.78 kg/m^2^. More pregnancies went to late term in the MMS group (*n* = 6) than in the placebo group (*n* = 3), and there was a higher rate of delivery complications within the MMS group (15.6%) versus the placebo group (3.0%), with no subsequent problems. 

### 3.2. Efficacy Endpoints

#### 3.2.1. Primary Endpoint

Milk DHA increased with MMS over the course of the study, from a mean of 0.25% ± 0.09% (range 0.12–0.57%) at Visit 2 to 0.35% ± 0.08% (0.18–0.49%) at Visit 4. In contrast, there was a decrease with placebo from 0.26% ± 0.12% (0.12–0.64%) at Visit 2 to 0.21% ± 0.08% (0.10–0.47%) at Visit 4. The LS mean difference in milk DHA (wt % total fatty acids (TFA)) between MMS and placebo from Visit 2 to Visit 4 was 0.15 (0.11–0.19) (*p* < 0.0001 in favor of MMS) (significant changes in Table 2, with full results in Supplementary Table S5).

#### 3.2.2. Secondary Maternal Endpoints

There were no significant changes in maternal weight, height, or BMI between groups during the study. There were significant changes in maternal blood biomarkers and milk nutrient composition with MMS versus placebo, as outlined in Table 2. In line with the changes observed in milk, there was an increase in blood DHA with MMS from Visit 2 to Visit 4, compared with a decrease in the placebo group. There was also a significant increase in the blood and milk levels of the omega-3 fatty acid eicosapentaenoic acid (EPA) in the MMS group, but a decrease in the placebo group. The blood levels of the omega-6 fatty acid docosatetraenoic acid decreased in both groups, but the reduction was significantly greater with MMS than placebo.

The serum vitamin concentrations also differed significantly between groups. There were increases in the blood levels of 25-OH-vitamin D, folic acid, vitamin B12, and lutein with MMS compared to a decrease with placebo. Levels of beta carotene increased in blood and milk with MMS but decreased with placebo. In the MMS group, there was a decrease in blood homocysteine levels compared to an increase with placebo. Apart from these parameters, there were no significant differences in any other maternal blood biomarkers or the milk nutrient composition.

Assessment of nutritional status (FFQ) (Supplementary Table S6) showed that macro- and micro-nutrient intake from food was not always adequate. In particular, the mean values for energy, calcium, and iodine were lower than the RDA in both groups at Visits 2 and 4, while the mean zinc intake was lower in the placebo group at Visit 4. The lower range values in both groups also indicated inadequate intakes in some women for carbohydrate, fiber, protein, copper, iron, magnesium, manganese, sodium, zinc, vitamins A, D, C, and E, vitamins B1, B2, B3, B6, and B12, and folate. From Visit 2 to Visit 4, iodine intake decreased significantly in the MMS group compared with the placebo group (LS mean difference −47.02 (95% confidence interval (CI) −90.25, −3.79) μg; *p* = 0.0335). There were no other significant changes in FFQ parameters during the study.

The evaluation of wellbeing, using the fatigue questionnaire (MAF), showed no significant differences between groups from Visit 2 to Visit 4 (LS mean difference 3.07 (95% CI −1.867 to 7.998); *p* = 0.22).

#### 3.2.3. Exploratory Infant Endpoints

There were no significant differences in infant weight and length WHO z-score-for-age between groups at Visit 2 and 4 (Supplementary Table S7).

#### 3.2.4. Safety Analysis

During the study, no subject discontinued treatment and no mothers or infants died. One mother in the placebo group had a severe AE and three mothers in the MMS group had a serious AE (Table 3). Only one mother in each group experienced a treatment-related AE, considered mild intensity in the placebo group (flatulence) and moderate in the MMS group (peripartum hemorrhage).

One infant in the placebo group had a severe AE, while two in the MMS group and four in the placebo group experienced at least one serious adverse event (SAE). None of the AEs reported in infants were regarded as treatment related.

## 4. Discussion

Supplementation of healthy breastfeeding women from a high-income country with multiple nutrients including DHA and lutein resulted in increased maternal blood and milk concentrations of selected nutrients compared with placebo. A significant increase in milk DHA content was observed with supplementation. In contrast, there was a decrease with placebo. Similar results have been reported elsewhere, with DHA supplementation correlating with increased milk DHA levels [17,36]. The finding that DHA levels are preserved in breast milk after supplementation is important and may ultimately translate to clinical benefits in the infant [37]. Our results are also in agreement with a recent study in pregnant women [38], where there was an increase in maternal red blood cell levels of DHA after supplementation with multiple micronutrients and DHA, but a decrease in non-supplemented women. In our study, there was an overall increase in the milk and blood concentrations of the omega-3 long-chain polyunsaturated fatty acids (LCPUFAs) DHA and EPA (compared with a decrease with placebo) but a significantly greater decrease in the blood levels of omega-6 LCPUFA docosatetraenoic acid versus placebo. Although omega-6 LCPUFAs have essential roles in the regulation of inflammation, the eicosanoids derived from them are proinflammatory; in contrast, omega-3 eicosanoids have few inflammatory effects [39]. Our findings are in agreement with the observation that human milk fatty acid composition, and similarly plasma phospholipid fatty acids, are influenced by the dietary fatty acid intake [40]. Comparison of different populations indicates that milk DHA is highly influenced by maternal dietary DHA intake [41,42]. Although the contribution of fatty acids from tissue stores implies a time lag until the full effect of a fatty acid supplementation on milk composition is achieved, there is also direct transfer of fatty acids from diet to milk [18]. Thus, supplementation started during established lactation is effective and beneficial for the infant. It should be noted that the decrease in DHA levels in the placebo group may have occurred because during pregnancy, all women were allowed supplements that could have contained DHA. However, after birth and during lactation (our study period), supplements containing DHA were not permitted in the placebo group (as per the inclusion/exclusion criteria), which may have led to the decrease in DHA levels. Furthermore, the DHA content of human milk is known to decrease with increasing duration of lactation [17,43], presumably due to progressing depletion of maternal DHA body stores which contribute considerably to delivery of DHA for incorporation into human milk lipids [18,44,45].

As was to be expected, given that this study was performed in a high-income country, many women had optimal intake of most macro- and micro-nutrients. However, the FFQ indicated inadequate intakes of energy, calcium, and iodine (Supplementary Table S6). There were also indications of insufficient intakes for carbohydrate, fiber, protein, copper, iron, zinc, magnesium, manganese, sodium, zinc, vitamins A, D, C, and E, vitamins B1, B2, B3, B6, and B12, and folate. Nutrient inadequacies can have adverse effects in the body, such as impaired immunity, and should be addressed [46,47]. It is common for women of child-bearing age to have insufficient levels of vitamin D [48]. The problem is exacerbated in lactating women, as additional vitamin D is required in breastmilk for the child. Mean dietary intake of vitamin D in the women participating in our study was below dietary recommendations. It is important that infants receive enough vitamin D to promote bone mineralization and prevent rickets [49]. Early-life vitamin D has also been proposed to decrease susceptibility to infections and autoimmune disorders, but conclusive evidence is lacking [8,50]. Many infants and children do not achieve an adequate intake and status of vitamin D [6,51]. The desirable reference for serum 25-OH-vitamin D is 20–80 ng/mL [52]. In our study, mean baseline maternal blood levels of 25-OH-vitamin D were below the minimum value in both groups. In the placebo group, 25-OH-vitamin D concentrations fell even further during the course of the study. In the supplementation group, however, mean blood levels were within the range of desirable values at Visit 3 (mean 28.3 ng/mL) and remained at a similar level at Visit 4 (mean 29.6 ng/mL). A similar increase was observed in a study of breastfeeding women supplemented with 5000 IU/day (as opposed to the 600 IU/day in our study), where maternal serum 25-OH-vitamin D levels rose from 28.8 ng/mL at baseline to 43.9 ng/mL after 28 days [53]. Such an increase might confer benefits to the child, as observed by Oberhelman et al. [53]; nevertheless, it is recommended that all breastfed babies receive daily vitamin D (400 IU) supplementation, even if the mothers themselves take a supplement [54,55,56].

Similarly, we observed a significant increase in blood levels of lutein during our study, whereas levels fell in the placebo group. Our results are in line with those reported in maternal plasma concentrations after supplementation with lutein in lactating women [57]. The authors concluded that lactating women are highly responsive to lutein supplementation, which affects plasma lutein concentrations in the infant. However, milk levels of lutein did not increase after supplementation in our study (but also did not decrease, as observed with placebo). This may be for several reasons, such as the study being insufficiently powered to detect any significant increases in milk lutein, the concentration of lutein in the supplement being too low, or the duration of supplementation being too short.

Higher than normal levels of blood homocysteine resulting from low intakes of vitamins B6 and B12 and folate are considered a risk indicator for developing thrombotic and cardiovascular events in adults [58,59]. In our study, supplementation led to a significant increase in blood concentrations of folic acid and vitamin B12, along with decreasing blood homocysteine; in contrast, these vitamins decreased, and homocysteine increased in the placebo group.

Supplementation also increased blood and milk concentrations of beta-carotene, which decreased in the placebo group. A similar finding was reported in a study of beta-carotene supplementation in healthy lactating mothers [60], with increasing maternal serum and milk beta-carotene concentrations, but unchanged serum and milk retinol levels. The authors also found that infant serum retinol significantly increased without a change in beta-carotene in infant serum, indicating conversion of beta-carotene to retinol in the infant. Vitamin A has vital roles in mammary gland metabolism and adequate milk production, as well as during the weaning process [61]. Furthermore, breast milk is the only source of vitamin A for infants, and it is important to ensure they receive a sufficient intake. Another study observed that supplementation of the maternal diet with beta-carotene from the first trimester of gestation until three months after birth was associated with a small but negative effect on infant birth weight (−18 g, *p* = 0.06) [62]. We found no significant effects on infant growth in our study, although it was not powered to detect growth effects.

Supplementation with multiple micronutrients, lutein, and DHA was well-tolerated, and no treatment-emergent adverse effects were detected.

One of the strengths of this study is its randomized, placebo-controlled design, with comprehensive data from dietary intake, milk, and blood. Potential limitations include the limited sample size and the small diversity of participating women in terms of ethnicity, which could have impacted upon the natural nutrient composition of breast milk prior to supplementation. In addition, there was no evaluation of potential confounders. Further analysis on the composition of breast milk after supplementation would also be helpful, to help clarify whether increases in the maternal blood levels of micronutrients translates to increased levels in breastmilk, and thus increased availability to infants. The study was also set up to exploratorily analyze infant outcomes, but with a two-week window allowed for study visits. Although this window may be negligible when evaluating the effects of supplementation on biomarkers over time, it is not suitable when looking at infant anthropometrics, given that these change so rapidly during early life. Finally, it is important to evaluate the effects of maternal nutritional status on relevant clinical outcomes in the mother and baby. Thus, adequately powered, longer-term studies are necessary to provide further information on the clinical impact of the nutrient status effects observed in the women on both maternal health and infant outcomes, which have previously been suggested [63,64,65].

## 5. Conclusions

Maternal dietary supplementation with multiple micronutrients, lutein, and DHA during the lactating period significantly increased maternal milk DHA content compared with placebo, without any indications of adverse effects. In this population of women with no overt nutritional deficiencies, macro- and micro-nutrient intake from food was often insufficient. Supplementation led to significant increases in maternal blood levels of DHA and EPA, 25-OH-vitamin D, folate, vitamin B12, lutein, and beta-carotene. Supplementation also led to a significant decrease in homocysteine.

## Figures and Tables

**Table 1:** Subject characteristics at baseline and delivery information (per protocol population).

Characteristics	Placebo (*n* = 33)	MMS (*n* = 32)	Total (*n* = 65)
Age, years	31.7 ± 4.0 (21–38)	31.7 ± 3.5 (25–39)	31.7 ± 3.8 (21–39)
Race:			
Caucasian	33 (100.0)	28 (87.5)	61 (93.8)
Black/African American	0	1 (3.1)	1 (1.5)
Asian	0	2 (6.3)	2 (3.1)
Other	0	1 (3.1)	1 (1.5)
Weight, kg	70.8 ± 10.29 (52.0–110.0)	69.3 ± 13.30 (52.2–107.0)	70.1 ± 11.80 (52.0–110.0)
Height, cm	1.67 ± 0.08 (1.48–1.90)	1.68 ± 0.06 (1.53–1.83)	1.67 ± 0.07 (1.48–1.90)
Body mass index, kg/m^2^	25.29 ± 3.62 (19.8–38.1)	24.6 ± 3.97 (19.3–32.4)	25.0 ± 3.78 (19.3–38.1)
Gestational age, weeks: ^1^			
Early term	6 (18.2)	5 (15.6)	11 (16.9)
Full term	24 (72.7)	21 (65.6)	45 (69.2)
Late term	3 (9.1)	6 (18.8)	9 (13.8)
Delivery type:			
Vaginal	27 (81.8)	24 (75.0)	51 (78.5)
Caesarean	6 (18.2)	8 (25.0)	14 (21.5)
Delivery complications, Yes	1 (3.0)	5 (15.6)	6 (9.2)
Infant birth weight, g	3400.6 ± 355.6 (2860–4130)	3556.9 ± 347.5 (2856–4375)	3477.6 ± 357.7 (2856–4375)

All values expressed as mean ± standard deviation (range) or *n* (%), as appropriate. ^1^ Early term, ≥37 to <39 weeks; full term, ≥39 to <41 weeks; late term, ≥41 to <42 weeks. MMS, multiple micronutrients, lutein, and docosahexaenoic acid supplementation.

**Table 2:** Primary and secondary maternal efficacy endpoints with significant changes reported from Visit 2 to Visit 4 (per protocol population, LOCF approach).

Parameters	Placebo (*n* = 33)	MMS (*n* = 32)	LS Mean Difference (95% CI) ^1^	*p* Value *
	Mean ± SD (Range)			
Milk parameters:				
DHA (wt % TFA) (primary)	−0.05 ± 0.11 (−0.32 to 0.22)	0.11 ± 0.12 (−0.23 to 0.32)	0.15 (0.11–0.19)	<0.0001
EPA, %	−0.01 ± 0.04 (−0.11 to 0.06)	0.01 ± 0.03 (−0.06 to 0.06)	0.0110 (0.0006–0.0214)	0.038
Mead acid, %	0.019 ± 0.054 (−0.03 to 0.19)	−0.004 ± 0.015 (−0.07 to 0.01)	−0.022 (−0.040 to −0.003)	0.024
Beta carotene, ng/mL	−5.1 ± 17.2 (−48.0 to 28.0)	22.7 ± 34.5 (−20.0 to 142.0)	28.4 (15.0–41.9)	<0.0001
Blood parameters:				
DHA, mg/L	−9.76 ± 8.98 (−37.0 to 6.9)	7.14 ± 11.40 (−29.0 to 28.1)	15.66 (11.96–19.36)	<0.0001
DHA/TFA	−0.004 ± 0.007 (−0.008 to −0.001)	0.009 ± 0.009 (−0.02 to 0.03)	0.013 (0.010–0.016)	<0.0001
Docosatetraenoic acid, mg/L	−0.44 ± 1.08 (−3.9 to 1.9)	−0.70 ± 0.87 (−2.2 to 1.8)	−0.46 (−0.86 to −0.05)	0.0270
EPA, mg/L	−2.84 ± 4.27 (−11.2 to 4.6)	0.59 ± 4.48 (−10.6 to 13.5)	2.21 (0.44–3.98)	0.0155
25-OH-vitamin D, ng/mL	−1.02 ± 8.18 (−7.5 to 5.50)	5.88 ± 9.98 (−8.4 to 28.9)	7.82 (4.36–11.28)	<0.0001
Folic acid, ng/mL	−1.73 ± 4.73 (−11.6 to 16.5)	17.82 ± 9.51 (11.70−23.80)	21.20 (17.84–24.56)	<0.0001
Homocysteine, μM	0.56 ± 1.47 (−3.51 to 3.72)	−1.08 ± 1.24 (−4.24 to 1.71)	−1.63 (−2.27 to −0.99)	<0.0001
Vitamin B12, pg/mL	−17.9 ± 97.0 (−313.0 to 342.0)	69.0 ± 135.7 (−190.0 to 510.0)	89.89 (31.45–148.3)	0.0031
Beta carotene, ng/mL	−49.3 ± 160.3 (−443.0 to 222.0)	223.3 ± 334.2 (−480.0 to 1182.0)	296.35 (183.06–409.64)	<0.0001
Lutein, ng/mL	−16.8 ± 33.1 (−91.0 to 40.0)	5.8 ± 52.2 (−182.0 to 90.0)	21.13 (4.15–38.31)	0.0157

* *p* value < 0.05 considered statistically significant. ^1^ Difference = supplementation – placebo. CI, confidence interval; DHA, docosahexaenoic acid; EPA, eicosapentaenoic acid; LOCF, last observation carried forward; LS mean, least squares mean; MMS, multiple micronutrients, lutein, and DHA supplementation; SD, standard deviation; TFA, total fatty acids; wt, weight.

**Table 3:** Summary of participants who experienced treatment-emergent adverse events (AEs) and suspected treatment-related AEs (safety population; values expressed as *n* (%) subjects).

Parameters	Placebo (*n* = 35)	MMS (*n* = 35)
Mothers with at least one AE, *n*	19	18
Mothers with at least one AE: ^1^		
Mild	11 (57.9)	10 (55.6)
Moderate	7 (36.8)	8 (44.4)
Severe	1 (5.3)	0
Mothers with at least one SAE	0	3 (16.7)
Mothers with at least one treatment-related AE ^2^	1 (5.3)	1 (5.6)
Number of infant AEs, *n*	9	7
Infants with at least one AE: ^1^		
Mild	2 (28.6)	6 (66.7)
Moderate	4 (57.1)	3 (33.3)
Severe	1 (14.3)	0
Infants with at least one SAE	4 (57.1)	2 (22.2)
Infants with at least one treatment-related AE	0	0

NB. AEs are defined as any untoward medical occurrence in a clinical investigation subject administered a pharmaceutical product and which does not necessarily have to have a causal relationship with this treatment. The term “severe” is often used to describe the intensity (severity) of a specific event (as in mild, moderate, or severe myocardial infarction); the event itself, however, may be of relatively minor medical significance (such as headache). This is not the same as “serious,” which is based on patient/event outcome or action criteria usually associated with events that pose a threat to a patient’s life or functioning. Seriousness (not severity) serves as a guide for defining regulatory reporting obligations. ^1^ The majority of AEs were classified in the SOC ‘infections and infestations’, with nasopharyngitis being the most commonly reported AE. ^2^ For the two mothers who experienced treatment-emergent AEs, one was classified under the SOC ‘gastrointestinal disorders’ and the PT ‘flatulence’ (placebo) and one was classified under the SOC ‘pregnancy, puerperium, and perinatal conditions’ and the PT ‘peripartum hemorrhage’ (MMS). AE, adverse event; MMS, multiple micronutrients, lutein, and docosahexaenoic acid supplementation; PT, preferred terms; SAE, serious adverse event; SOC, system organ class.

## Supplementary Materials

The following are available online at https://www.mdpi.com/2072-6643/12/12/3849/s1, Table S1: Assessment schedule, Table S2: Full list of inclusion and exclusion criteria, Table S3: Composition of the multiple micronutrients, lutein, and DHA supplement (MMS) compared with the daily dietary intake recommended for lactating women by the Institute of Medicine (IOM), Table S4: Efficacy parameters assessed during the trial at V2, V3 and V4, Table S5: Primary and secondary maternal efficacy endpoints with changes reported from V2 to V4 (per protocol population, LOCF approach)., Table S6: Results of the food frequency questionnaire at V2 and V4 (per protocol population), with comparison to recommended dietary allowance (RDA). Values in bold fall below the RDA, Table S7: Exploratory infant parameters: weight and length as well as weight/length WHO z-score-for-age values at V2 and V4 and regression analysis a (per protocol population).
