# Supplementary material for: Multiple Micronutrients, Lutein, and Docosahexaenoic Acid Supplementation during Lactation: A Randomized Controlled Trial
Source: Nutrients. 2020 Dec 16;12(12):3849. doi: 10.3390/nu12123849 (PMC7765837; doi:10.3390/nu12123849)
Supplement: Supplementary file 1 [file nutrients-12-03849-s001.pdf]

Supplementary tables

Table 1. Assessment schedule.

Visit and timing	V1a	V1b	V2 B&R	PC1	PC2	V3	PC3	PC4	V4 EoS	PC5 Final FU
	Screening									
	GA week 34/35	2–4 wks after delivery	4–6 wks after delivery	2 wks after V2 (±3 days)	4 wks after V2 (±3 days)	6 wks after V2 (±3 days)	8 wks after V2 (±3 days)	10 wks after V2 (±3 days)	12 wks after V2 (±3 days)	7 days after V4 (±3 days)
Subject informed consent	X									
Inclusion/exclusion criteria	X	X	X							
Medical/surgical history	X									
Demographics	X									
History of previous birth outcomes	X									
History or current intake of lutein, DHA, and MMN intake	X	X	X			X			X	
History and review of drug, alcohol and nicotine use	X	X	X			X			X	
Assessment of pregnancy	X									
Medication history over past 30 days	X									
Physical examination	X	X	X			X			X	
Vital signs (sitting blood pressure, pulse rate)	X	X	X			X			X	
Height, weight and body mass index [a]	X	X	X			X			X	
Type of delivery		X								
Infant birth weight and infant assessment		X								
Record infant Apgar score at 5 min after birth		X								
Delivery complications		X								
Blood sampling for fatty acids in plasma GPL, DHA/TFA ratio, IFN-gamma; TGF-beta, folic acid, vitamin B12 and homocysteine, alpha-tocopherol and retinol, carotenoids (lutein, zeaxanthin, lycopene, carotene), 25(OH)D			X			X			X	

Visit and timing	V1a	V1b	V2 B&R	PC1	PC2	V3	PC3	PC4	V4 EoS	PC5 Final FU
	Screening									
	GA week 34/35	2–4 wks after delivery	4–6 wks after delivery	2 wks after V2 (±3 days)	4 wks after V2 (±3 days)	6 wks after V2 (±3 days)	8 wks after V2 (±3 days)	10 wks after V2 (±3 days)	12 wks after V2 (±3 days)	7 days after V4 (±3 days)
Mothers' milk nutritional composition [b]			X			X			X	
Randomization of trial product kit to subject			X							
Check for supplementation compliance and breastfeeding status				X	X	X	X	X	X	
Return of study product						X			X	
Dispense new study product			X			X			X	
Review of concomitant medications		X	X	X	X	X	X	X	X	
Food consumption assessment (Food Frequency Questionnaire)	X		X			X			X	
Well-being assessment (psychological fatigue questionnaire)			X			X			X	
Infant assessments [c]			X			X			X	
Assess adverse events		X	X	X	X	X	X	X	X	X
Safety laboratory	X [d]	X	X			X [e]			X	
Subject identification assignment	X									
Subject discontinuation									X	

[a] Body mass index was assessed only at V1b, 2, and 4. [b] Mothers' baseline milk nutritional composition assessment included: total lipid DHA content, total lipids, DHA/total FA ratio, milk macronutrient content, IFN-gamma, TGF-beta, alpha-tocopherol, retinol, carotenoids (lutein, zeaxanthin, beta-cryptoxanthin, lycopene, carotene. [c] Infant assessments included: weight, length, body composition (calculation of weight and length standard deviation scores (SDS), infant feeding history and status, infant health assessment. [d] At V1, additional samples for hepatitis and human immunodeficiency virus I and II serology. [e] At V3, the safety assessments were limited to activated partial thromboplastin time (aPTT), prothrombin time (PT) and international normalized ratio (INR). 25(OH)D, 25-hydroxyvitamin D; B, baseline; DHA, docosahexaenoic acid; EoS, end of study; FU, follow-up; GA, gestational age; GPL, glycerophospholipids; IFN, interferon gamma; MMN, multiple micronutrients; PC, phone call; R, randomization; TFA, total fatty acids; TGF, transforming growth factor; V, visit; wks, weeks.

Table 2. Full list of inclusion and exclusion criteria.

Inclusion criteria	**Women:**<br>1. Healthy pregnant women aged 18–45 years (inclusive) in their third trimester of pregnancy who are expected to give birth to a healthy single full-term infant<br>2. Hemoglobin >105 g/L<br>3. Intention to breastfeed for at least four months (no more than one bottle or 10% of total milk intake daily as formula)<br>4. Omnivorous diet<br>5. No intent to take multivitamin supplements, DHA supplements, lutein supplements or any combination of the aforementioned after giving birth except for iodine and iron<br>6. Seronegative for human immunodeficiency virus, hepatitis B, and hepatitis C at screening<br>7. Pregnant women who, in the opinion of the Investigator, were willing and able to participate in all scheduled visits, to adhere to the study plan, laboratory tests, and all other study related procedures according to the clinical protocol<br>8. Pregnant women who provided a personally signed and dated informed consent willing to participate in the study and to adhere to all study procedures including the assessments done to the infant, indicating that they have been informed of all pertinent aspects of the trial and that they understood and accepted these, prior to admission to the study<br>**Infants:**<br>1. Full term/gestational age >37 weeks <43 weeks and birth weight adequate for gestational age<br>2. Apgar score at 5 minutes after birth >7<br>3. No indication of abnormal neurodevelopment
Exclusion criteria	**Women:**<br>1. Physical (including vital signs, e.g., blood pressure, pulse rate), hematological and clinical-chemical parameters deviating from normal and with clinical relevance in the opinion of the investigator<br>2. Any serious infection (acute or chronic) at screening and randomization<br>3. Any history of or current metabolic diseases (e.g., diabetes, hypothyroidism, and other metabolic diseases)<br>4. Less than 12 months from previous delivery<br>5. Any history of or current diseases associated with malabsorption, or other severe diseases of the gastrointestinal tract (e.g. chronic inflammatory bowel disease, iron accumulation, iron utilization disorders)<br>6. Any history of or current neurological, cardiac, endocrine or bleeding disorders<br>7. Specific diets (e.g., vegan, vegetarian, celiac)<br>8. Pre-pregnancy body mass index <18.5 or >30 kg/m^2<br>9. Diagnosed or suspected malignant or premalignant disease<br>10. Current clinically significant depression<br>11. Not willing, or unable for medical reasons to interrupt any intake of pharmaceuticals or dietary supplements which may interact with any of the ingredients of the trial product (i.e. fluoroquinolones, bisphosphonates, levodopa, levothyroxine, penicillamine, antibiotics containing tetracycline or trietine) during the study supplementation period (from V2 (baseline and randomization) until V4 (end of study)<br>12. History of or current diseases where vitamin, mineral, trace element, lutein, or DHA supplementation might be not recommended/contraindicated (such as sickle cell anemia, copper metabolism disorders (Wilson's disease), renal disease,

nephrolithiasis, urolithiasis, hypercalcemia, hypercalciuria, hepatobiliary diseases, existing hypervitaminosis, iron metabolism disorders, hypermagnesemia)

13. Any pregnancy complications or adverse pregnancy outcomes in current pregnancy that may affect micronutrient metabolism or status (e.g. preeclampsia, eclampsia, polyhydramniosis, placental insufficiency)
14. Diagnosed congenital abnormalities in current pregnancy
15. Known carrier or affected with a genetic disease or condition (e.g. mutation carrier for autosomal recessive diseases)
16. History of or current abuse of drugs, alcohol or other substances
17. Current smoker or smoker during current pregnancy
18. Any history of hypersensitivity or known allergy to any of the ingredients of the study supplement
19. Incapability of understanding the language in which the study related information was given
20. Close affiliation with the investigational site, e.g., a close relative of the investigator, dependent person (e.g. employee of the investigational site)
21. Unwilling or unable to comply with all requirements outlined in the protocol
22. Women previously screened into the study could not be re-included
23. Women who were currently participating in a clinical study or who took part in another clinical study during the course of present pregnancy (including nutritional studies and non-interventional studies)

Infants:

1. Congenital anomalies
2. Obvious gastrointestinal or metabolic disorders
3. Perinatal hypoxia
4. Preterm birth
5. Very low birth weight (small for gestational age)

DHA, docosahexaenoic acid; V, visit.

Table 3. Composition of the multiple micronutrients, lutein, and DHA supplement (MMS) compared with the daily dietary intake recommended for lactating women by the Institute of Medicine (IOM) [1].

Micronutrient, units	MMS soft gel capsule	IOM [1] [1]
Vitamin A, IU	3600	4333 (3999)
Vitamin C, mg	60	120 (115)
Vitamin D, IU	600	600
Vitamin E, IU	10	28
Vitamin B1, mg	1.4	1.4
Vitamin B2, mg	1.6	1.6
Vitamin B3, mg	17	17
Vitamin B5, mg	7	7
Vitamin B6, mg	2	2
Vitamin B12, µg	2.8	2.8
Folic acid, µg	500	500
Biotin, µg	35	35 [2]
Calcium, mg	120	1000 (1300)
Iodine, µg	225	290
Iron, mg	9	9 (10)
Selenium, µg	55	70
Zinc, mg	10	12 (13)
DHA, mg	200	250 + 100–200 [3]
Lutein, µg	250	ND

[1] Specific recommendation for lactating women, value in parentheses indicates specific recommendation for lactating women <19 years old; [2] Average intake; [3] European Food Safety Authority recommendation of 250 mg/day eicosapentaenoic acid + DHA plus 100–200 mg/day DHA for pregnant and lactating women [2]. DHA, docosahexaenoic acid; ND, not determined.

Table 4. Efficacy parameters assessed during the trial at V2, V3 and V4.

Efficacy parameters	Sampling method
**Maternal blood biomarkers**	
FA in GPL	GC
DHA/TFA ratio	GC
IFN-gamma	ELISA
TGF-beta	ELISA
Folic acid	ECL
Vitamin B12	ECL
Homocysteine	LC-MS
25(OH)D	ECL
Alpha tocopherol and retinol	HPLC
Carotenoids (lutein, zeaxanthin, beta-cryptoxanthin, lycopene, carotene)	HPLC
**Maternal milk composition**	GC
Total lipids	GC
%DHA/TFA ratio	GC
Milk macronutrient content	Spectroscopy
IFN-gamma	ELISA
TGF-beta	ELISA
Alpha tocopherol and retinol	HPLC
Carotenoids (lutein, zeaxanthin, beta-cryptoxanthin, lycopene, carotene)	HPLC
**Other maternal variables**	
Weight, height and body mass index	—
Wellbeing/Fatigue	Validated multidimensional assessment of fatigue (MAF) questionnaire [3,4]
Nutritional status	Food frequency questionnaire [5] [a]
**Exploratory variables**	
Infant weight and length	—
Infant feeding	—
Infant health history	—
Body composition (SDS)	—

[a] Focus on foods providing lutein and DHA. 25(OH)D, 25-hydroxyvitamin D; DHA, docosahexaenoic acid; ECL, electro-chemiluminescence immunoassay; ELISA, enzyme-linked immunosorbent assay; FA, fatty acids; GC, gas chromatography; GPL, glycerophospholipids; HPLC, high-performance liquid chromatography; IFN, interferon; LC-MS, liquid chromatography-mass spectrometry; SDS, calculation of weight and length standard deviation scores; TFA, total fatty acids; TGF, transforming growth factor; V, visit.

Table 5. Primary and secondary maternal efficacy endpoints with changes reported from V2 to V4 (per protocol population, LOCF approach).

Parameters	Placebo (*n* = 33) Mean±SD (range)	MMS (*n* = 32) Mean±SD (range)	LS mean difference (95% CI) [1]	*P* value *
**Milk parameters:**				
DHA (wt% TFA) (primary)	−0.05±0.11 (−0.32 to 0.22)	0.11±0.12 (−0.23 to 0.32)	0.15 (0.11–0.19)	**<0.0001**
ALA, %	−0.08±0.54 (−1.44 to 1.28)	−0.07±045 (−1.38 to 1.28)	−0.10 (−0.30 to 0.09)	0.3064
Arachidonic acid, %	−0.01±0.07 (−0.14 to 0.26)	−0.05±0.07 (−0.24 to 0.09)	−0.02 (−0.06 to 0.01)	0.1710
Decanoic acid, %	−0.11±0.38 (−0.83 to 0.54)	−0.11±0.47 (−1.42 to 0.55)	−0.0007 (−0.1667 to 0.1653)	0.9934
DGLA, %	−0.07±0.08 (−0.38 to 0.10)	−0.08±0.08 (−0.38 to 0.10)	−0.02 (−0.05 to 0.01)	0.2086
Docosanoic acid, %	−0.011±0.089 (−0.22 to 0.15)	0.002±0.076 (−0.22 to 0.14)	−0.004 (−0.037 to 0.030)	0.8293
DPA (n−3), %	0.006±0.065 (−0.09 to 0.32)	−0.004±0.041 (−0.18 to 0.06)	−0.011 (−0.035 to 0.012)	0.3333
DPA (n−6), %	−0.007±0.035 (−0.10 to 0.09)	0.008±0.045 (−0.08 to 0.16)	0.015 (−0.00009 to 0.02993)	0.0514
Docosatetraenoic acid, %	−0.001±0.022 (−0.03 to 0.07)	−0.012±0.017 (−0.07 to 0.01)	−0.0086 (−0.0177 to 0.0005)	0.0634
Eicosadienoic acid, %	−0.04±0.06 (−0.19 to 0.09)	−0.03±0.05 (−0.14 to 0.09)	0.006 (−0.016 to 0.028)	0.5808
EPA, %	0.01±0.04 (−0.11 to 0.06)	0.01±0.03 (−0.06 to 0.06)	0.0110 (0.0006–0.0214)	**0.0380**
Eicosatrienoic acid, %	−0.012±0.015 (−0.04 to 0.02)	−0.006±0.016 (−0.04 to 0.04)	−0.00003 (−0.00599 to 0.00594)	0.9933
Eicosenoic acid, %	−0.05±0.09 (−0.28 to 0.16)	−0.03±0.09 (−0.17 to 0.22)	0.002 (−0.037 to 0.042)	0.9045
GLA, %	−0.006±0.043 (−0.10 to 0.11)	−0.016±0.029 (−0.08 to 0.03)	−0.012 (−0.028 to 0.004)	0.1429
Heptadecanoic acid, %	0.011±0.057 (−0.11 to 0.12)	0.008±0.056 (−0.13 to 0.16)	−0.003 (−0.026 to 0.021)	0.8224
Linoleic acid, %	0.09±2.98 (−8.32 to 5.81)	0.27±3.05 (−6.27 to 10.32)	0.26 (−0.96 to 1.49)	0.6672
Mead acid, %	0.019±0.054 (−0.03 to 0.19)	−0.004±0.015 (−0.07 to 0.01)	−0.022 (−0.040 to −0.003)	**0.0243**
Lauric acid, %	−0.06±1.83 (−3.51 to 4.65)	−0.01±2.32 (−6.37 to 5.28)	0.06 (−0.82 to 0.95)	0.8866
Myristic acid, %	0.41±1.98 (−3.21 to 6.00)	0.25±2.36 (−4.57 to 5.26)	−0.11 (−1.12 to 0.89)	0.8248
Myristoleic acid, %	−0.005±0.100 (−0.23 to 0.18)	−0.008±0.099 (−0.26 to 0.20)	−0.002 (−0.045 to 0.040)	0.9099
Octanoic acid, %	0.009±0.121 (−0.12 to 0.59)	−0.018±0.093 (−0.24 to 0.18)	−0.024 (−0.071 to 0.024)	0.3191
Oleic acid, %	−0.25±3.65 (−6.19 to 7.28)	−0.11±4.00 (−9.36 to 7.55)	−0.05 (−1.52 to 1.42)	0.9436
Palmitic acid, %	0.15±2.61 (−6.39 to 4.44)	0.37±2.71 (−6.37 to 4.28)	−0.29 (−1.39 to 0.81)	0.6001

Parameters	Placebo (*n* = 33)	MMS (*n* = 32)	LS mean difference (95% CI) [1]	*P* value *
	Mean±SD (range)			
Palmitoleic acid, %	−0.009±0.598 (−1.00 to 2.02)	−0.139±0.728 (−1.98 to 1.45)	−0.0006 (−2.8666 to 0.2854)	0.9967
Pentadecanoic acid, %	−0.035±0.106 (−0.13 to 0.24)	−0.005±0.116 (−0.32 to 0.23)	−0.035 (−0.086 to 0.0157)	0.1710
Stearic acid, %	0.10±2.64 (−7.40 to 6.22)	0.47±2.06 (−3.87 to 8.62)	0.01 (−0.84 to 0.87)	0.9792
Tetracosaenoic acid, %	−0.006±0.025 (−0.05 to 0.08)	−0.009±0.025 (−0.11 to 0.03)	−0.005 (−0.015 to 0.006)	0.3752
Trans−docosenoic acid, %	−0.003±0.036 (−0.09 to 0.11)	−0.004±0.027 (−0.06 to 0.09)	−0.008 (−0.012 to 0.005)	0.2319
Trans−palmitoleic acid, %	0.005±0.023 (−0.04 to 0.06)	−0.001±0.021 (−0.06 to 0.04)	−0.004 (−0.013 to 0.005)	0.3896
Trans/trans−linoleic acid, %	0.021±0.052 (−0.05 to 0.19)	0.004±0.043 (−0.13 to 0.08)	−0.015 (−0.004 to 0.007)	0.1813
Transoctadecenoic acid, %	−0.05±0.16 (−0.55 to 0.13)	0.01±0.10 (−0.36 to 0.29)	0.02 (−0.01 to 0.06)	0.1706
Tridecanoic acid, %	0.012±0.028 (−0.04 to 0.09)	0.004±0.022 (−0.04 to 0.07)	−0.010 (−0.021 to 0.001)	0.0773
Vaccenic acid, %	0.05±0.27 (−0.46 to 0.58)	−0.07±0.30 (−0.75 to 0.55)	−0.01 (−0.14 to 0.11)	0.8400
Total lipids – M1, g/100 mL	−0.59±1.71 (−4.5 to 2.5)	−0.16±2.05 (−4.0 to 5.8)	0.43 (−0.28 to 1.15)	0.2315
Total lipids – M2, g/100 mL	−0.57±1.97 (−4.2 to 3.4)	−0.25±2.29 (−4.2 to 6.2)	0.53 (−0.28 to 1.35)	0.1976
Total lipids – mean, g/100 mL	−0.58±1.82 (−4.4 to 2.7)	−0.21±2.16 (−4.0 to 6.0)	0.48 (−0.28 to 1.24)	0.2082
TGF−b2, pg/mL	249.5±3246.3 (−6760 to 15375)	460.3±4436.3 (−13966 to 15655)	717.13 (−749.52 to 2183.78)	0.3322
Alpha tocopherol, µg/mL	−0.80±2.71 (−7.39 to 4.13)	−0.64±2.78 (−5.30 to 6.82)	0.32 (−0.70 to 1.35)	0.5315
Vitamin A (retinol), µg/mL	−0.17±0.42 (−1.23 to 0.76)	−0.16±0.38 (−0.91 to 0.79)	0.02 (−0.12 to 0.16)	0.7285
Lutein, ng/mL	−5.0±21.2 (−44.0 to 41.0)	0.1±24.4 (−47.0 to 57.0)	5.7 (−4.4 to 15.8)	0.2636
Beta carotene, ng/mL	−5.1±17.2 (−48.0 to 28.0)	22.7±34.5 (−20.0 to 142.0)	28.4 (15.0–41.9)	**<0.0001**
Beta−cryptoxanthin, ng/mL	−7.0±13.0 (−43.0 to 18.0)	−3.5±14.5 (−35.0 to 33.0)	2.2 (−2.7 to 7.2)	0.3757
Lycopen, ng/mL	−0.8±9.8 (−19.0 to 22.0)	−2.7±8.7 (−19.0 to 19.0)	0.4 (−3.4 to 4.1)	0.8398
Zeaxanthin, ng/mL	−1.9±7.8 (−23.0 to 13.0)	−0.1±9.0 (−18.0 to 34.0)	1.2 (−2.3 to 4.7)	0.4944
Carbohydrate – M1, g/100 mL	−0.16±0.39 (−1.1 to 0.5)	−0.21±0.89 (−4.6 to 0.5)	−0.10 (−0.42 to 0.21)	0.5203
Carbohydrate – M2, g/100 mL	−0.12±0.39 (−1.0 to 0.6)	−0.21±0.85 (−4.3 to 0.7)	−0.12 (−0.41 to 0.18)	0.4247
Carbohydrate – Mean, g/100 mL	−0.14±0.38 (−1.0 to 0.5)	−0.21±0.87 (−4.5 to 0.5)	−0.11 (−0.42 to 0.19)	0.4717

Parameters	Placebo (*n* = 33)	MMS (*n* = 32)	LS mean difference (95% CI) [1]	*P* value *
	Mean±SD (range)			
Energy – M1, g/100 mL	−7.1±16.1 (−44.0 to 20.0)	−2.2±19.8 (−41.0 to 60.0)	4.67 (−2.36 to 11.70)	0.1891
Energy – M2, g/100 mL	−6.9±19.6 (−42.0 to 30.0)	−3.4±21.9 (−45.0 to 65.0)	5.46 (−2.48 to 13.40)	0.1744
Energy – Mean, g/100 mL	−7.0±17.2 (−43.0 to 24.0)	−2.8±20.7 (−41.0 to 63.0)	5.06 (−2.36 to 12.48)	0.1776
Protein (crude) – M1, g/100 mL	−0.23±0.27 (−1.3 to 0.2)	−0.04±1.01 (−1.0 to 5.3)	0.21 (−0.14 to 0.57)	0.2377
Protein (crude) – M2, g/100 mL	−0.23±0.23 (−0.9 to 0.3)	−0.04±1.03 (−0.5 to 5.5)	0.22 (−0.15 to 0.59)	0.2350
Protein (crude) – Mean, g/100 mL	−0.23±0.24 (−1.1 to 0.2)	−0.04±1.01 (−0.8 to 5.4)	0.22 (−0.14 to 0.58)	0.2318
Protein (true) – M1, g/100 mL	−0.18±0.23 (−1.0 to 0.2)	−0.03±0.82 (−0.8 to 4.3)	0.17 (−0.12 to 0.46)	0.2454
Protein (true) – M2, g/100 mL	−0.18±0.20 (−0.7 to 0.2)	−0.01±0.86 (−0.5 to 4.6)	0.20 (−0.11 to 0.51)	0.2050
Protein (true) – Mean, g/100 mL	−0.18±0.20 (−0.9 to 0.2)	−0.02±0.83 (−0.6 to 4.5)	0.18 (−0.12 to 0.48)	0.2239
TS – M1, g/100 mL	−0.96±1.81 (−5.0 to 2.1)	−0.40±2.21 (−5.1 to 6.5)	0.55 (−0.26 to 1.35)	0.1804
TS – M2, g/100 mL	−0.92±2.07 (−4.7 to 2.8)	−0.51±2.43 (−5.8 to 7.5)	0.65 (−0.26 to 1.55)	0.1587
TS – Mean, g/100 mL	−0.94±1.92 (−4.9 to 2.3)	−0.46±2.31 (−5.3 to 7.0)	0.60 (−0.25 to 1.44)	0.1649
**Blood parameters:**				
ALA, mg/L	−0.89±1.75 (−6.21 to 2.0)	−0.33±2.17 (−6.4 to 5.9)	0.16 (−0.63 to 0.94)	0.6902
Arachidonic acid, mg/L	−13.70±20.57 (−55.9 to 26.7)	−15.48±15.20 (−38.8 to 18.5)	−3.46 (−10.78 to 3.86)	0.3487
DGLA, mg/L	−7.72±10.17 (−41.8 to 10.4)	−5.45±10.44 (−24.2 to 21.9)	−0.26 (−4.87 to 4.35)	0.9113
DHA, mg/L	−9.76±8.98 (−37.0 to 6.9)	7.14±11.40 (−29.0 to 28.1)	15.66 (11.96–19.36)	**<0.0001**
DPA (n−3), mg/L	−1.24±2.51 (−7.8 to 2.8)	−0.94±2.62 (−6.3 to 7.4)	−0.53 (−1.66 to 0.59)	0.34555
DPA (n−6), mg/L	−1.11±1.23 (−4.4 to 0.5)	−0.83±1.28 (−4.6 to 1.6)	0.01 (−0.39 to 0.42)	0.9412
Docosatetraenoic acid, mg/L	−0.44±1.08 (−3.9 to 1.9)	−0.70±0.87 (−2.2 to 1.8)	−0.46 (−0.86 to −0.05)	**0.0270**
Eicosadienoic acid, mg/L	−0.92±1.07 (−3.5 to 1.4)	−0.68±0.91 (−2.4 to 1.6)	−0.007 (−0.366 to 0.352)	0.9691
Eicosanoic acid, mg/L	−0.37±0.87 (−3.3 to 1.2)	−0.28±0.82 (−2.2 to 1.2)	0.08 (−0.30 to 0.46)	0.6774
EPA, mg/L	−2.84±4.27 (−11.2 to 4.6)	0.59±4.48 (−10.6 to 13.5)	2.21 (0.44–3.98)	**0.0155**
Eicosatrienoic acid (n−3), mg/L	−0.42±0.62 (−1.6 to 0.6)	−0.33±0.52 (−1.6 to 1.3)	0.05 (−0.22 to 0.31)	0.7212
Eicosenoic acid, mg/L	−0.24±0.52 (−1.3 to 0.9)	−0.19±0.43 (−1.1 to 0.9)	−0.03 (−0.24 to 0.19)	0.7892

Parameters	Placebo (*n* = 33)	MMS (*n* = 32)	LS mean difference (95% CI) [1]	*P* value *
	Mean±SD (range)			
GLA, mg/L	−0.28±0.98 (−2.7 to 2.1)	−0.5±0.69 (−1.5 to 2.1)	−0.10 (−0.41 to 0.22)	0.5445
Heptadecanoic acid, mg/L	−0.49±0.86 (−2.2 to 1.6)	−0.40±0.69 (−1.9 to 0.9)	−0.02 (−0.34 to 0.29)	0.8908
Linoleic acid, mg/L	−31.26±61.87 (−193.1 to 80.3)	−25.11±44.50 (−118.0 to 68.0)	−2.42 (−22.90 to 18.05)	0.8137
Mead acid, mg/L	−0.72±1.20 (−3.0 to 2.4)	−0.49±0.99 (−2.1 to 1.9)	0.03 (−0.41 to 0.47)	0.8865
Myristic acid, mg/L	−1.14±2.16 (−7.2 to 2.8)	−0.69±3.26 (−6.6 to 8.6)	0.06 (−1.03 to 1.15)	0.9108
Myristoleic acid, mg/L	−0.06±0.22 (−0.6 to 0.4)	0.02±0.29 (−0.7 to 0.5)	0.02 (−0.09 to 0.13)	0.7206
Oleic acid, mg/L	−18.69±33.19 (−113.3 to 35.5)	−12.07±25.96 (−56.1 to 86.1)	−1.07 (−13.07 to 10.94)	0.8598
Palmitic acid, mg/L	−2.01±3.03 (−9.2 to 3.9)	−1.84±2.79 (−7.7 to 6.2)	−0.04 (−1.28 to 1.20)	0.9495
Pentadecanoic acid, mg/L	−0.15±0.28 (−1.0 to 0.4)	−0.13±0.32 (−1.3 to 0.4)	−0.02 (−0.14 to 0.10)	0.7132
Stearic acid, mg/L	−27.15±38.37 (−119.7 to 33.2)	−8.88±28.76 (−64.3 to 42.4)	9.33 (−4.14 to 22.80)	0.1712
Trans−docosenoic acid, mg/L	−0.71±1.67 (−6.3 to 2.8)	−0.10±1.74 (−5.6 to 2.7)	0.47 (−0.19 to 1.12)	0.1598
Trans−palmitoleic acid, mg/L	−0.08±0.12 (−0.3 to 0.2)	−0.02±0.15 (−0.4 to 0.3)	0.06 (−0.01 to 0.13)	0.0942
Trans/trans−linoleic acid, mg/L	−0.06±0.39 (−0.9 to 1.2)	0.10±0.99 (−1.3 to 5.0)	0.16 (−0.20 to 0.51)	0.3898
Transoctadecenoic acid, mg/L	−0.15±0.55 (−1.1 to 1.5)	−0.12±0.45 (−1.2 to 0.8)	−0.04 (−0.24 to 0.17)	0.7138
Vaccenic acid, mg/L	−2.43±2.57 (−7.6 to 3.1)	−2.57±2.48 (−7.7 to 3.2)	−0.30 (−1.39 to 0.78)	0.5912x
DHA/TFA	−0.004±0.007 (−0.008 to −0.001)	0.009±0.009 (−0.02 to 0.03)	0.013 (0.010–0.016)	**<0.0001**
TFA, mg/L	−180.98±207.73 (−638.5 to 151.8)	−109.22±152.81 (−419.5 to 297.6)	23.17 (−48.07 to 94.41)	0.5180
IFN−γ, pg/mL	−1.42±3.32 (−11.1 to 1.2)	−0.69±3.19 (−11.8 to 2.3)	0.44 (−0.40 to 1.29)	0.3000
TGF−b2, pg/mL	42.78±200.07 (−571.9 to 483.4)	25.63±327.89 (−386.3 to 1100.6)	38.37 (−104.67 to 181.40)	0.5889
Folic acid, ng/mL	−1.73±4.73 (−11.6 to 16.5)	17.82±9.51 (11.70–23.80)	21.20 (17.84–24.56)	**<0.0001**
Vitamin B12, pg/mL	−17.9±97.0 (−313.0 to 342.0)	69.0±135.7 (−190.0 to 510.0)	89.89 (31.45–148.3)	**0.0031**
Homocysteine A2, µM	0.56±1.47 (−3.51 to 3.72)	−1.08±1.24 (−4.24 to 1.71)	−1.63 (−2.27 to −0.99)	**<0.0001**
25−OH−vitamin D, ng/mL	−1.02±8.18 (−7.5 to 5.50)	5.88±9.98 (−8.4 to 28.9)	7.82 (4.36–11.28)	**<0.0001**
Alpha−tocopherol, µg/mL	−2.37±2.32 (−8.5 to 2.0)	−1.31±1.86 (−6.8 to 2.2)	0.61 (−0.22 to 1.43)	0.4151

Parameters	Placebo (*n* = 33)	MMS (*n* = 32)	LS mean difference (95% CI) [1]	*P* value *
	Mean±SD (range)			
Vitamin A (retinol), μg/mL	−0.03±0.09 (−0.22 to 0.18	0.02±0.10 (−0.18 to 0.36)	0.03 (−0.01 to 0.047)	0.1222
Beta carotene, ng/mL	−49.3±160.3 (−443.0 to 222.0)	223.3±334.2 (−480.0 to 1182.0)	296.35 (183.06–409.64)	**<0.0001**
Beta−cryptoxanthin, ng/mL	−47.1±84.1 (−312.0 to 15.0)	−28.8±63.4 (−206.0 to 66.0)	4.32 (−11.44 to 20.08)	0.5856
Lutein, ng/mL	−16.8±33.1 (−91.0 to 40.0)	5.8±52.2 (−182.0 to 90.0)	21.13 (4.15–38.31)	**0.0157**
Lycopen, ng/mL	−22.5±106.6 (−353.0 to 242.0)	−65.9±145.9 (−472.0 to 192)	−17.26 (−57.29 to 22.76)	0.3919
Zeaxanthin, ng/mL	−4.9±11.1 (−31.0 to 13.0)	−1.5±16.5 (−55.0 to 30.0)	2.65 (−2.45 to 7.76)	0.3026

* Significant differences (i.e., p<0.05; two-sided) indicated in bold. ALA, alpha linoleic acid; DGLA, dihomo-gamma-linoleic acid; DHA, docosahexaenoic acid; DPA, docosapentaenoic acid; GLA, gamma linoleic acid; IFN-γ, interferon gamma; LS, least squares; MMS, multiple micronutrients, lutein, and DHA supplement; SD, standard deviation; TFA, total fatty acids; TGF, transforming growth factor; TS, total carbohydrate solids; V, visit.

Table S6. Results of the food frequency questionnaire at V2 and V4 (per protocol population), with comparison to recommended dietary allowance (RDA). Values in bold fall below the RDA.

**Daily intake**	**Placebo (*n* = 33), mean±SD (range)**		**MMS (*n* = 32), mean±SD (range)**		**IOM [1] [a]**
	**V2**	**V4**	**V2**	**V4**	
Energy, kcal	**2108.9±697.5** (**1279.0**–3915.0)	**1929.6±645.4** (**1029.0**–3733.0)	**2377.6±1035.2** (**882.0**–5053.0)	**2066.7±747.5** (**949.0**–4066.0)	2733–2803 (2698–2768)
Carbohydrate – total, g	266.7±89.1 (**165.0**–496.0)	249.7±87.9 (**145.0**–518.0)	306.1±145.4 (**81.0**–680.0)	268.6±104.9 (**109.0**–548.0)	210
Cholesterol, mg	257.1±104.4 (110.0–484.0)	228.9±81.2 (101.0–382.0)	287.7±152.3 (119.0–781.0)	243.8±89.6 (113.0–528.0)	ALAP
Englyst fiber – NSP, g	29.5±12.2 (**12.6**–60.1)	**27.6±11.1** (**12.8**–57.0)	34.4±19.9 (**6.5**–105.1)	31.2±15.0 (**10.2**–75.7)	29 [b]
Fat – total, g	72.2±27.5 (36.0–135.0)	63.6±26.4 (29.0–127.0)	78.5±35.7 (31.0–179.0)	66.5±25.2 (31.0–139.0)	ND
Protein, g	77.6±26.4 (**42.8**–140.3)	**69.7±20.0** (**36.6**–113.1)	87.7±35.9 (**31.6**–203.2)	76.7±26.3 (**38.3**–138.8)	71
Alcohol, g	0.4±0.64 (0.0–2.6)	0.9±1.44 (0.0–5.6)	0.5±1.03 (0.0–5.2)	0.5±1.07 (0.0–5.6)	—
Calcium, mg	**685.9±253.2** (**380.0**–1322.0)	**597.2±231.4** (**212.0**–1122.0)	**778.8±378.3** (**259.0**–1993.0)	**658.5±281.8** (**233.0**–1233.0)	1000 (1300)
Chloride, mg	3702.1±1500.8 (**1589.0**–6479.0)	3517.0±1317.8 (**1274.0**–5706.0)	3772.1±1932.2 (**1433.0**–10391.0)	3445.0±1562.9 (**1245.0**–7727.0)	2300 [b]
Copper, µg	1866.2±596.8 (**922.0**–3223.0)	1779.5±581.0 (**1019.0**–3220)	2289.8±1020.3 (**866.0**–5242.0)	2035.2±805.9 (**1012.0**–4053.0)	1300
Iron, mg	22.2±17.9 (**7.4**–111.0)	19.1±9.6 (**6.0**–44.2)	44.5±77.5 (**5.8**–436.5)	58.9±184.4 (**6.7**–1042.4)	9 (10)
Iodine, µg	**173.3±93.1** (**47.0**–327.0)	**173.4±124.2** (**38.0**–594.0)	**226.8±120.8** (**39.0**–570.0)	**161.7±98.7** * (**50.0**–397.0)	290
Magnesium, mg	509.4±496.2 (**196.0**–2951)	439.0±169.3 (**185.0**–2884)	532.4±373.2 (**154.0**–2105.0)	416.3±239.1 (**162.0**–1320.0)	310–320 (360)
Manganese, µg	5706.8±2685.8 (**1761.0**–14061.0)	5219.8±2559.0 (**2369.0**–14001.0)	6469.3±3191.9 (**2160.0**–15052.0)	5476.1±2532.7 (**2294.0**–13965.0)	2600 [b]

Daily intake	Placebo (*n* = 33), mean±SD (range)		MMS (*n* = 32), mean±SD (range)		IOM [1] [a]
	V2	V4	V2	V4	
Sodium, mg	2102.7±806.8 (**952.0**–3547.0)	1974.0±712.8 (**721.0**–3156.0)	2159.7±1041.4 (**788.0**–5686.0)	1942.2±866.5 (**799.0**–4266.0)	1500 [b]
Phosphorus, mg	1224.4±423.3 (721.0–2458.0)	1104.3±328.4 (**605.0**–1976.0)	1387.7±583.4 (**529.0**–3101.0)	1230.0±446.2 (**609.0**–2171.0)	700 (1250)
Zinc, mg	12.2±4.3 (**6.3**–22.6)	**11.3±4.6** (**4.9**–26.4)	15.2±6.5 (**4.7**–31.1)	13.5±6.3 (**6.0**–28.5)	12 (13)
Vitamin A – retinol, mg	1.34±1.69 (**0.13**–6.23)	1.32±1.67 (**0.13**–6.16)	**0.99**±0.64 (**0.17**–2.29)	1.39±1.68 (**0.14**–6.07)	1.3 (1.2)
Vitamin C, mg	254.2±89.9 (**91.0**–406.0)	245.4±109.7 (**78.0**–458.0)	318.5±219.7 (**54.0**–1312.0)	290.6±169.1 (**29.0**–697.0)	120 (115)
Vitamin D – ergocalciferol, µg	**12.8±10.7** (**0.6**–33.7)	**13.2±15.5** (**0.4**–68.0)	**14.9±11.2** (**0.7**–42.2)	**11.3±11.1** (**0.7**–42.3)	15 [6]
Vitamin E – alpha tocopherol, mg	**15.8±6.8** (**5.9**–32.4)	**15.3±7.6** (**5.3**–33.9)	**17.0±7.0** (**5.1**–31.7)	**15.1±8.9** (**4.4**–40.4)	19
Vitamin B1, mg	2.93±1.81 (**0.82**–7.28)	2.88±1.89 (**0.61**–7.02)	3.37±1.93 (**0.72**–8.00)	2.73±2.04 (**0.72**–9.35)	1.4
Vitamin B2, mg	2.19±0.95 (**0.93**–5.52)	2.18±1.17 (**0.63**–5.98)	2.61±1.19 (**0.71**–6.07)	2.32±1.40 (**0.78**–6.18)	1.6
Vitamin B3, µg	39861.1±12284.3 (19426.0–70599.0)	38495.5±14109.1 (**15777.0**–72397.0)	45984.2±16574.9 (**13858.0**–96414.0)	39908.2±15564.7 (17538.0–69969.0)	17000
Vitamin B6, mg	3.87±2.24 (**1.10**–9.70)	3.93±2.44 (**0.86**–9.55)	4.50±2.85 (**1.00**–11.69)	3.68±2.79 (**1.03**–12.91)	2
Vitamin B12, µg	9.26±7.80 (**1.8**–29.8)	9.02±8.33 (**1.2**–30.8)	11.34±10.26 (**1.2**–43.4)	8.53±9.06 (**1.6**–41.2)	2.8
Folate – Total, µg	777.0±347.2 (**206.0**–1294.0)	810.0±561.4 (**168.0**–3034.0)	1080.9±584.4 (**176.0**–2215.0)	799.8±525.1 (**106.0**–2051.0)	500

* Significantly greater reduction in iodine intake from V2 to V4 with MMS vs. placebo (least squares mean difference −47.02 (95% confidence interval −90.25 to −3.79; p=0.0335).
[a] Specific recommendation for lactating women, value in parentheses indicates specific recommendation for lactating women <19 years old; [b] average intake. ALAP, as low as possible; IOM, institute of Medicine; MMS, multiple micronutrients, lutein, and DHA supplement; ND, not determined; NSP, non-starch polysaccharides; SD, standard deviation; V, visit.

Table S7. Exploratory infant parameters: weight and length as well as weight/length WHO z-score-for-age values at V2 and V4 and regression analysis [a] (per protocol population)

Infant parameters	Placebo (*n* = 33[a])	MMS (*n* = 32[b])	Difference at V4	
	Mean±SD		adj. difference with 95% CI [c]	*P* value [c]
Weight, kg				
V2	4.6±0.57	4.8±0.60		
V4	6.8±0.75	6.8±0.90	−0.16 (−0.35, 0.31)	0.069
WHO z-score weight-for-age				
V2	−0.10±0.85	0.02±0.75		
V4	−0.10±0.87	−0.15±1.07	−0.17 (−0.48, 0.13)	0.136
Length, cm				
V2	56.1±2.19	57.4±2.49		
V4	65.2±2.49	64.7±2.33	−1.29 (-3.31, 0.74)	0.111
WHO z-score length-for-age				
V2	0.47±1.08	0.92±1.11		
V4	1.01±1.13	0.55±0.93	−0.69 (−1.84, 0.47)	0.125

[a] *n* = 30 for z-score; [b] *n* = 31 for z-score; [c] coefficients (difference in MMS compared to placebo group), 95% confidence interval (CI) and *p*-value of respective outcome at V4 from robust regression with clustering on site with adjustment for respective anthropometric measure at V2, study site and sex; SD, standard deviation; V, visit.

References

1. Food Nutrition Board of the Institute of Medicine. Nutrient Recommendations: Dietary Reference Intakes (DRI). National Institutes of Health, Office of Dietary Supplements **2020**, https://ods.od.nih.gov/Health_Information/Dietary_Reference_Intakes.aspx. Date accessed: 1 Apr 2020.
2. Flock, M.R.; Harris, W.S.; Kris-Etherton, P.M. Long-chain omega-3 fatty acids: time to establish a dietary reference intake. *Nutr Rev* **2013**, *71*, 692-707.
3. Fairbrother, N.; Hutton, E.K.; Stoll, K.; Hall, W.; Kluka, S. Psychometric evaluation of the Multidimensional Assessment of Fatigue scale for use with pregnant and postpartum women. *Psychol Assess* **2008**, *20*, 150-158.
4. Wambach, K.A. Maternal fatigue in breastfeeding primiparae during the first nine weeks postpartum. *J Hum Lact* **1998**, *14*, 219-229.
5. Mulligan, A.A.; Luben, R.N.; Bhaniani, A.; Parry-Smith, D.J.; O'Connor, L.; Khawaja, A.P.; Forouhi, N.G.; Khaw, K.T. A new tool for converting food frequency questionnaire data into nutrient and food group values: FETA research methods and availability. *BMJ Open* **2014**, *4*, e004503.
6. IOM (Institute of Medicine). *Dietary reference intakes for calcium and vitamin D*. The National Academies Press: Washington DC, 2011.
